# Scarlet Fever

**Published:** 1901-01-05

**Authors:** Percival Mackie

**Affiliations:** Resident Medical Officer, Bristol City Isolation Hospitals, Ham Green


### HOSPITAL CLINICS AND MEDICAL PROGRESS.

### SCARLET FEVER.

By PERCIVAL MACKIE, M.R.C.S.Eng., L.R.C.P.Lond.; Resident Medical Officer, Bristol City Isolation Hospitals, Ham Green.

(*Concluded from page* 223.)

*Respiratory System.*—Ulceration rarely extends from the throat to the larynx, but œdema of the glottis is not an uncommon complication, and these cases are almost invariably fatal even when relieved by tracheotomy.

*Digestive System.*—The tongue in many cases possesses no special peculiarities. Most commonly it is covered with a thin layer of dirty-white fur, and the red papillæ-like points are visible through the fur. This is not peculiar to scarlet fever, as the red papillæ may be seen on any furred tongue if the "fur" is not too thick. In the characteristic scarlet-fever tongue—often talked about but rarely seen—the papillæ in the anterior two-thirds are enlarged sometimes to the size of the circumvallate papillæ, smooth and oval. This has been aptly called the "raspberry" tongue. The tongue of scarlet fever used to be called the "strawberry" tongue. Whether this applied to its "furred" or to its "clean" state we could never make out. In neither case is the simile a very apt one. The tongue is the first part of the body to desquamate if the disappearance of the fur in patches can be so termed. This is said to begin with great regularity on the fourth day; but in our experience, although this is frequently the case, it is not always so. Vomiting rarely persists. In one case, however, a child æt. seven years, it continued for three or four days resisting all treatment, and the patient died. At the post-mortem absolutely nothing was found to account for death.

*The Excretory System.*—The kidneys are particularly susceptible to the scarlet-fever poison. A form of acute nephritis may be met with during the first week of the fever which does not differ from that occurring in other conditions. It affects the tubules and has no special partiality for the glomeruli, and presents the usual symptoms of acute tubal nephritis. The only case we have seen proved rapidly fatal in about 24 hours from the onset. The special "scarlet fever kidney," however, comes on during the second, third or fourth week, most commonly the third, and is characterised by the almost constant presence of blood in the urine, and pathologically by the involvement of the glomeruli, at first entirely, and throughout to a greater extent than the other structures of the kidney. We have often been struck by the constancy of blood in the urine, even in very mild cases, so much so that one can pick out at once the specimens which contain albumen by their smoky or bloody colour. If there is any appreciable quantity of albumen there is almost certain to be blood also. In only one case, that of a child admitted with advanced nephritis, in the third week, has death followed, except in the instance mentioned above. In the other cases there was never either dropsy, uræmia, pericardial or pleuritic effusion or retinitis, nor in fact any of the complications which sometimes occur in renal disease; the mischief seemed to be entirely relegated to the kidneys themselves. None of the cases even excreted large quantities of albumen, rarely as much as 2 grammes per litre, and the amount of urine passed during the 24 hours was always nearly normal. The percentage of urea varied of course with the amount of urine passed, and was generally between 2 to 3 per cent. Microscopic examination of the deposit in acute cases shows abundance of granular and blood casts, blood cells and renal epithelium generally mived with amorphous urates. Two cases only have been discharged with still a trace of albumen, the rest completely recovered as far as one could tell.

*Other Complications.*—Arthritis or septic rheumatism (1·2 per cent.) is not a true rheumatic manifestation but a similar condition to that met with in pyæmia, gonorrhœa, and other microbial diseases. It affects the wrist-joints most frequently, then the ankles, knees, and less commonly other joints. It is erratic and wandering like true rheumatism and also resembles it in its external appearances of redness and heat. But these joint outbreaks are not associated with acid sweats nor with copious urates in the urine as met with in rheumatism. Certain other complications require mention.

*Otitis media* (8 per cent.) is most frequent in young children, and is nearly always followed by otorrhœa. The pain lasts a few hours only, and then the tympanum gives way and the pain subsides. Sudden alarming rises of temperature are not infrequent in cases with otorrhœa, due probably to retention of pus in the recesses of the middle ear. The temperature, which may be 104° or 105° drops as suddenly as it rose, and the otorrhœa recommences. The danger of otorrhœa is that it may lead to meningitis, sinus thrombosis and pyæmia, mastoid disease or cerebral abscess, and it may leave permanent deafness. One of this series of cases developed meningitis and died after having previously recovered from a very severe and long-continued attack of the anginose form of the disease. One case admitted said to have had scarlet fever died in coma the next day, and at the autopsy an encysted abscess, holding one ounce of thick pus was found in the left frontal lobe which, however, did not seem to be connected with the middle ear. Adenitis is met with at two stages of the disease, either primarily in connection with an anginose throat when in severe cases it tends to go on to cervical cellulitis, or, secondarily, during the third week when a gland, or mass of glands, suddenly become enlarged quite apart from any severe condition. The temperature generally goes up to 103°, or more, and sometimes the gland goes on to suppuration. We have noticed as a point of clinical interest that very often together with this special adenitis there is a slight amount of blood and albumen in the urine. Whether there is any connection between the two facts we cannot say, but the association is frequent enough to strike the attention forcibly.

